# Progesterone Induced Blocking Factor Reduces Hypertension and Placental Mitochondrial Dysfunction in Response to sFlt-1 during Pregnancy

**DOI:** 10.3390/cells10112817

**Published:** 2021-10-20

**Authors:** Evangeline Deer, Jalisa Jones, Denise C. Cornelius, Kyleigh Comley, Owen Herrock, Nathan Campbell, Sarah Fitzgerald, Tarek Ibrahim, Babbette LaMarca, Lorena M. Amaral

**Affiliations:** 1 Department of Pharmacology and Toxicology, University of Mississippi Medical Center, Jackson, MS 39216, USA; edeer@umc.edu (E.D.); jonespjalisa@gmail.com (J.J.); kmcomley@go.olemiss.edu (K.C.); oherrock@umc.edu (O.H.); ncampbell@umc.edu (N.C.); sjfitzgerald@umc.edu (S.F.); ibrahimt@uthscsa.edu (T.I.); bblamarca@umc.edu (B.L.); 2 Department of Emergency Medicine, University of Mississippi Medical Center, Jackson, MS 39126, USA; dcornelius@umc.edu; 3 Department of Obstetrics and Gynecology, University of Mississippi Medical Center, Jackson, MS 39216, USA

**Keywords:** hypertension, preeclampsia, sFlt-1, oxidative stress, placental ischemia

## Abstract

Preeclampsia (PE) is characterized by new onset hypertension in association with placental ischemia, reduced fetal weight, elevated soluble fms-like tyrosine kinase-1 (sFlt-1), and placental mitochondrial (mt) dysfunction and oxidative stress (ROS). Progesterone induced blocking factor (PIBF) is a product of progesterone signaling that blocks inflammatory processes and we have previously shown PIBF to lower mean arterial blood pressure (MAP) and sFlt-1 in a rat model of PE. Infusion of sFlt-1 causes hypertension and many characteristics of PE in pregnant rodents, however, its role in causing mt dysfunction is unknown. Therefore, we hypothesize that PIBF will improve mt function and MAP in response to elevated sFlt-1 during pregnancy. We tested our hypothesis by infusing sFlt-1 via miniosmotic pumps in normal pregnant (NP) Sprague-Dawley rats (3.7 μg·kg^−1^·day^−1^) on gestation days (GD) 13–19 in the presence or absence of PIBF (2.0 µg/mL) injected intraperitoneally on GD 15 and examined mean arterial blood pressure (MAP) and placental mt ROS on GD 19. sFlt-1 increased MAP to 112 + 2 (n = 11) compared to NP rats (98 + 2 mmHg, n = 15, *p* < 0.05), which was lowered in the presence of sFlt-1 (100 + 1 mmHg, n = 5, *p* < 0.05). Placental mtATP was reduced in sFlt-1 infused rats versus NP controls, but was improved with PIBF. Placental mtROS was elevated with sFlt-1 compared to NP controls, but was reduced with PIBF. Sera from NP + sFlt-1 increased endothelial cell mtROS, which was attenuated with PIBF. These data demonstrate sFlt-1 induced HTN during pregnancy reduces placental mt function. Importantly, PIBF improved placental mt function and HTN, indicating the efficacy of improved progesterone signaling as potential therapeutics for PE.

## 1. Introduction

Preeclampsia (PE) is defined as new onset hypertension during pregnancy after the 20th week of gestation. It is associated with or without chronic immune activation, proteinuria, fetal growth restriction, and maternal endothelial dysfunction [1]. PE is a major cause of maternal, fetal, and neonatal morbidity and mortality affecting 5–7% of pregnancies worldwide [2]. Unfortunately, pregnant women with PE are at a higher risk of developing severe complications such as placental abruption, cerebrovascular dysfunction, multiorgan dysfunction, and disseminated intravascular coagulation. The fetus is at risk for intrauterine growth restriction, prematurity, and intrauterine death [3]. Despite being a leading cause of maternal death and maternal and perinatal morbidity, the mechanisms responsible for the pathogenesis of PE are unclear. Therefore, additional investigation into the pathophysiological mechanisms resulting in the development of PE is needed in order to elucidate potential therapies.

In PE, hypertension develops during the late second or third trimesters of pregnancy and remits after delivery in most cases, thereby implicating the placenta as the primary culprit in the disease. Studies have indicated that inflammatory cytokines increase and that there is an imbalance of regulatory and effector T cells and reactive oxygen species. Studies have implicated an imbalance in angiogenic factors, such as increased soluble fms-like tyrosine kinase 1 (sFlt-1) and decreased placental and vascular endothelial growth factor. These factors have been shown to mediate endothelial dysfunction causing the release of endothelin-1 (ET-1) and a decrease of vasodilators, such as nitric oxide, which contributes to the development of hypertension [4,5,6,7,8,9,10,11,12]. Increased sFlt-1 is known to decrease renal function and cause hypertension in non-pregnant women and during pregnancy [13]. Additionally, circulating and placental levels of sFlt-1 mRNA have been notably higher in women who have PE compared to normal pregnant women [14]. Tam Tam et al. demonstrated that increases in blood pressure in response to chronic sFlt-1 in pregnant rats is associated with increases in ROS in the placenta, kidney, and aorta, which was attenuated with Tempol, demonstrating that ROS is an important mediator of hypertension in response to sFlt-1 during pregnancy [15]. Although many studies demonstrate the importance of sFlt-1 to cause detrimental effects to the fetus and maternal hypertension during pregnancy, we do not know if mt dysfunction is an addition mechanism compromised by elevated sFlt-1 during PE.

Oxidative stress is an imbalance between reactive oxygen species (ROS) and antioxidant defense in the cell and occurs in normal pregnancies, however, overproduction of ROS leads to a decline of antioxidants, resulting in adverse pregnancy outcomes [16,17,18]. ROS have a major role in cell physiology as second messengers in signaling pathways, but also have a central role in the initiation of placental and endothelial dysfunction [16]. A central function of mitochondria (mt) is to produce energy in the form of adenosine triphosphate (ATP) making mt integral to cell survival. Changes in mitochondrial metabolic and bioenergetics, such as elevations in mt ROS and a decrease in respiration, can cause mitochondrial structural changes or death [19]. This decrease in ATP and electron transport activity (ETC) reduces important cellular functions, leading to tissue damage and a change in organ homeostasis [20]. Our previous studies have demonstrated a role for mitochondrial dysfunction and oxidative stress in the kidney and placenta of the pregnant RUPP rat model of PE or in response to RUPP CD4+ T cells or TNF-α during pregnancy, indicating an important role for inflammation to mediate multi-organ mt ROS and dysfunction in association with hypertension in pregnant rats [21,22,23,24].

A substantial amount of evidence indicates that progesterone may balance the inflammatory environment in the early trimesters of pregnancy [25]. In the presence of progesterone, lymphocytes release a protein known as progesterone induced blocking factor (PIBF), which mediates anti-inflammatory effects [26,27,28]. Previously, we have shown that circulating progesterone is decreased in PE patients and that progesterone decreased ET-1 secretion in vascular endothelial cells treated with serum from preeclamptic patients [29]. We have shown that PIBF improves inflammatory cytokines and decreases NK cell activation and CD4+ T cells in association with lowered ET-1 and sFLT-1 and, thus, hypertension in RUPP rats [30]. Importantly, PIBF normalized signs of PE, such as sFlt-1, which is known to play a role in hypertension and reducing fetal weight in PE [14,31]. However, it is unknown if PIBF can affect the role of mt mediated ROS in response to sFlt-1 induced hypertension during pregnancy.

## 2. Materials and Methods

### 2.1. Animals

Pregnant female Sprague Dawley (SD) rats weighing approximately 200–250 g were purchased from Envigo (Indianapolis, IN, USA) for use in the study. Rats were housed in a temperature-controlled room (23 °C) with a 12:12 hour light/dark cycle with free access to standard rat chow and water. The experiments were in compliance with the guidelines of the University of Mississippi Medical Center, and the animals were handled based on the principles in the National Institutes of Health Guide for the Care of Animals and the Institutional Animal Care and Use Committee (IACUC). 

### 2.2. Infusion of sFlt-1/PIBF into Pregnant Sprague Dawley Rats

Rats were divided into two control groups, consisting of normal pregnant (NP, n = 10) and sFlt-1 induced hypertensive rats (NP+ sFlt-1, n = 9). On gestational day (GD) 13, sFlt-1 was infused into NP Sprague Dawley rats (3.7 μg·kg^−1^·day^−1^) for 6 days via mini-osmotic pumps (model 2001, Alzet Scientific Corporation, Cupertino, CA, USA) as previously described (11). PIBF (2.0 µg/mL) (NP + PIBF) (n = 9) was injected intraperitoneally on GD 15 to sFlt-1 induced hypertensive pregnant rats. On day 18, indwelling carotid catheters were inserted in all groups for blood pressure measurements. Using isoflurane anesthesia (Webster, Sterling, MA, USA), catheters inserted were V3 tubing (Scientific Commodities, Inc., Lake Havasu City, AZ, USA), and were tunneled to the back of the neck and exteriorized. Carprofen (5 mg/kg) was administered via subcutaneous injection immediately following surgical procedure. On GD 19, blood pressure was measured with a pressure transducer (Cobe II tranducer CDX Sema, Aurora, CO, USA) and recorded continuously for one-hour after a 30 min stabilization period as previously described (11). Subsequently, placental and fetal weights were measured and blood and placentas were collected for mitochondrial function analysis.

### 2.3. Isolation of Mitochondria

Placental mitochondria were isolated using the differential centrifugation method [21,32]. Tissues were rinsed and homogenized using a Dounce homogenizer. The homogenate was centrifuged at 4000 rpm for 3 min at 4 °C. The supernatant was centrifuged at 10,000 rpm for 10 min at 4 °C, and the pellet was collected, suspended in 1 mL of Mito I buffer (250 mM sucrose, 10 mM HEPES, 1 mM EGTA 0.1% BSA, pH 7.2), and centrifuged at 10,000 rpm for 10 min at 4 °C. Afterwards, the pellet was suspended in 1 mL of Mito II (250 mM sucrose, 10 mM HEPES, 0.1% BSA, pH 7.2), and centrifuged at 10,000 rpm for 10 min at 4 °C. The final pellet was collected and suspended in 200 µL mL of Mito II buffer and used for respiration and ROS experiments.

### 2.4. Mitochondrial Respiration

Respiration in isolated placental mitochondrial was measured using the Oxgraph 2K (Oroboros Instruments, Innsbruck, Austria). The basal, state 2, state 3, state 4, and uncoupled respiration rates were measured using glutamate/malate, ADP, oligomycin, and FCCP (carbonyl cyanide-4-[trifluoromethoxy]phenylhydrazone), respectively [21]. Non-mitochondrial respiration was measured with the use of rotenone and antimycin A. The collected data were analyzed and expressed as pmol of oxygen consumed per second per milligram of mitochondrial protein. 

### 2.5. Mitochondrial ROS

Mitochondrial hydrogen oxygen peroxide (H_2_O_2_) production in placental mitochondria were determined using the amplex red assay [21,33]. Mitochondria (0.4 mg/mL) were incubated in a 96 well plate containing respiration buffer, superoxide dismutase (40 U/mL), horseradish peroxidase (4 U/mL), and succinate (10 mM). Amplex red (10 µM) was added to the wells last to initiate the reaction. The real-time production of H_2_O_2_ was measured using a plate reader at 555/581 nm excitation/emission for 30 min at 25 °C. Sample controls (blanks without amplex red or mitochondrial protein) were included in the assay.

### 2.6. Endothelial Mitochondrial ROS

MitoSOX red, a fluorogenic dye that targets the mitochondria in live cells, was used to measure mitochondrial specific reactive oxygen species. To determine vascular endothelial cell mt function, human umbilical venous endothelial cells (HUVECs) (ATCC) were grown in HUVEC media (Medium 199-DMEM (50:50), 10% FBS, and 1% antimycotic/antibiotic) to a 70% confluence on gelatin coated T25 culture flasks in a humidified atmosphere of 5% CO_2_ at 37 °C. At passage 4, the HUVEC cells were cultured in 6-well plates and incubated overnight. Cells were serum starved for 4h and incubated with 10% experimental NP serum (n = 6), NP + sFlt-1 serum (n = 5), or NP + sFlt-1+ PIBF serum (n = 5) overnight. Our lab has established that there is a significant increase in HUVEC mtROS after incubation with 10% RUPP rat serum [21]. After overnight incubation, experimental serums were washed off and cells were incubated with MitoSOX red (5 µM) for 30 min at 37 °C. Antimycin A (100 µM) was used as a positive control for the experiment. After washing the cells with DPBS twice, serum free medium was added and the cells were incubated for 4 hours. In the final stage, cells were collected and analyzed using flow cytometry in the FL2 channel (Miltenyi MACSQuant Analyzer 10, San Diego, CA, USA).

### 2.7. Statistical Analysis

Statistical analyses were performed using GraphPad Prism 7.02 software (GraphPad Software, San Diego, CA, USA). A one-way ANOVA with Bonferroni multiple comparisons test as post-hoc analysis were conducted for normally distributed variables. The Mann-Whitney U test or Kruskal–Wallis one-way ANOVA with Dunn’s multiple comparison post-hoc test were used for comparison of non-normally distributed variables. Results were reported as means ± SEM and were considered as statistically significant when *p* < 0.05

## 3. Results

**Figure 1:**
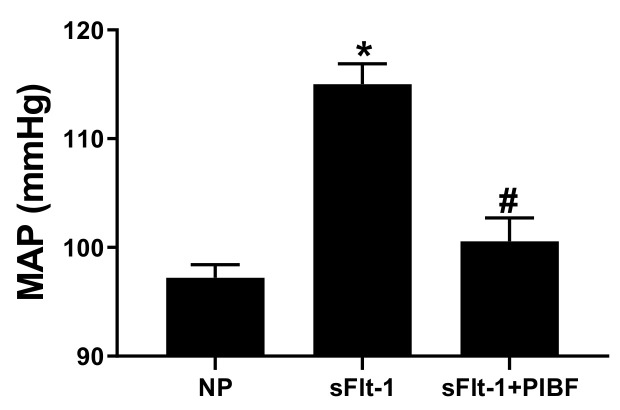
Infusion of sFlt-1 into NP rats increased MAP (n = 9) when compared with NP rats (n = 10, *p* < 0.05). Treatment with PIBF in the presence of sFlt-1 reduced MAP (n = 9, *p* < 0.05). Statistical differences were established using a one-way ANOVA. Results were reported as means ± SEM and considered statistically significant when *p* < 0.05 with using one-ANOVA. * *p* < 0.05 vs. NP control; # *p* < 0.05 vs. sFlt-1.

Mean arterial pressure (MAP) was increased in sFlt-1 infused rats to 112 ± 2 (n = 9) compared to control NP rats 98 ± 2 mmHg (n = 10, *p* < 0.05), Figure 1. Administration of PIBF reduced MAP to 100 ± 1 in the presence of sFlt1 (n = 9, *p* < 0.05). There were no significant changed in the body weights, placental weights, and fetal weights (Table 1).

**Figure 2:**
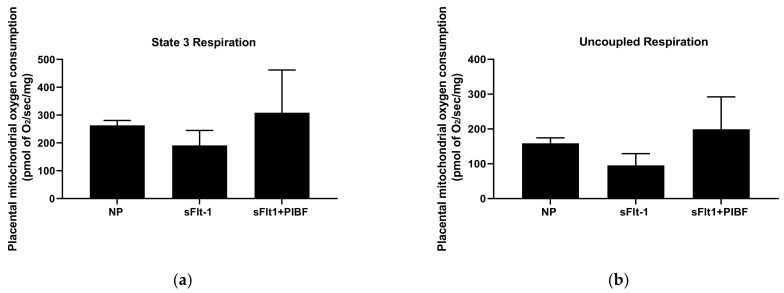
(**a**) State 3 placental mitochondrial respiration was reduced in placentas of sFlt-1 (n = 5) infused rats when compared to NP controls (n = 4), but was improved with PIBF (n = 5). (**b**) Uncoupled placental mitochondrial respiration was reduced in placentas of sFlt-1 infused rats (n = 5) when compared to NP controls (n = 4), but was improved with PIBF (n = 5). Statistical differences were established using a Kruskal–Wallis one-way ANOVA with Dunn’s multiple comparison post-hoc test. Results were reported as means ± SEM and considered statistically significant when *p* < 0.05.

Although not significant, placental mitochondrial state 3 respiration was lower in NP + sFlt-1 infused rats (191 ± 54 pmol of O2/s/mg, n = 5) compared to NP controls (263 ± 18 pmol of of O2/s/mg, n = 4) and was normalized in NP + sFlt-1+PIBF (309 ± 153 pmol of O2/s/mg, n = 5), (Figure 2A). Uncoupled respiration also decreased in NP + sFlt-1 infused rats (96 ± 34 pmol of O2/s/mg, n = 5) compared to NP controls (159 ± 16 pmol of O2/s/mg, n = 4) and was normalized with PIBF (199 ± 93 pmol of O2/s/mg, n = 5) (Figure 2B).

**Figure 3:**
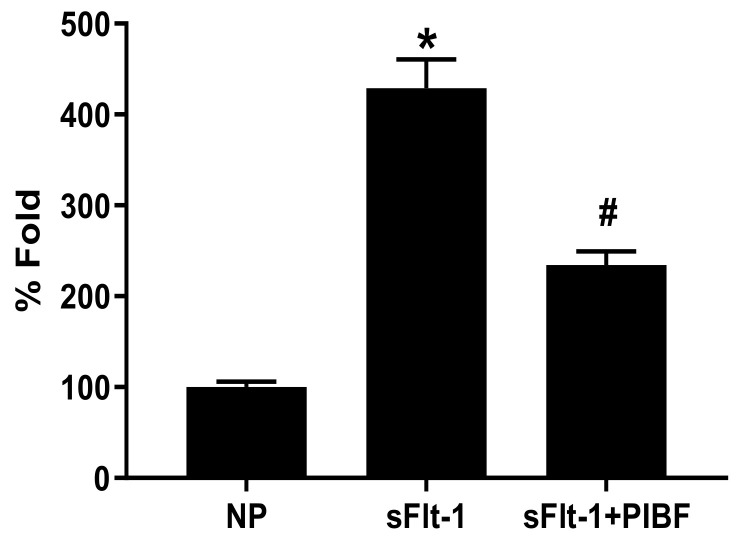
Mitochondrial ROS was significantly elevated in NP + sFlt-1 (n = 5, *p* < 0.05) but was lowered in both NP + sFlt-1+ PIBF (n = 5, *p* < 0.05) and NP controls (n = 5). Statistical differences were established using a Kruskal–Wallis one-way ANOVA with Dunn’s multiple comparison post-hoc test. Results were reported as means ± SEM and considered statistically significant when *p* < 0.05. * *p* < 0.05 vs. NP control; # *p* < 0.05 vs. sFlt-1 Kruskal–Wallis.

**Figure 4:**
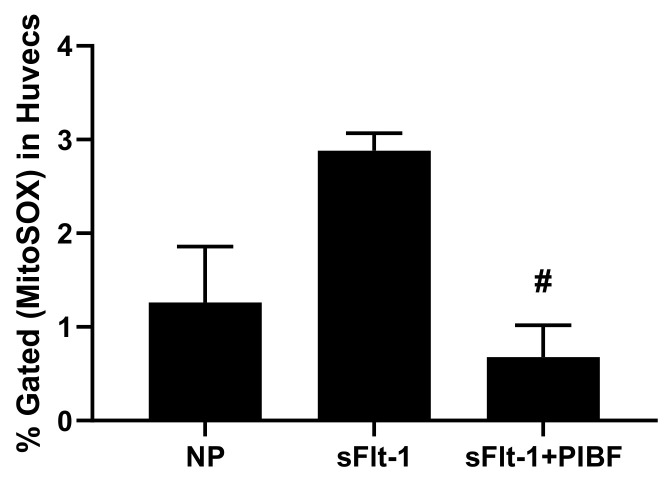
Sera from NP + sFlt-1 supplemented with PIBF attenuated endothelial cell mitochondrial ROS (n = 5, *p* < 0.05) compared to sera from NP + sFlt-1 (n = 5). Also, endothelial mt function was significantly reduced in NP + sFLT-1 compared to NP controls (n = 6). Statistical differences were established using a Kruskal–Wallis one-way ANOVA with Dunn’s multiple comparison post-hoc test. Results were reported as means ± SEM and considered statistically significant when *p* < 0.05. # *p* < 0.05 vs. sFlt-1 Kruskal–Wallis.

Moreover, placental mitochondrial ROS in NP + sFlt-1 was 429 ± 32% fold (n = 5, *p* < 0.05) compared to NP controls (100 ± 6% fold, n = 5), but was normalized in NP + sFlt-1 + PIBF (234 ± 15% fold, n = 5, *p* < 0.05) (Figure 3). Additionally, HUVECs incubated with 10% sera from NP + sFlt-1 rats exhibited increased maximal respiration (2.88 ± 0.18% gated, n = 5) compared to HUVECS treated with NP sera (1.26 ± 0.59% gated, n = 6), but HUVECS treated with sera from NP + sFlt-1 with PIBF was normalized (0.68 ± 0.34% gated, n = 5, *p* < 0.05) as shown in Figure 4.

### Figures, Tables, and Schemes

**Table 1:** Body, placental, and fetal weights.

	Body Weights (g)	Placental Weights (g)	Fetal Weights (g)
**NP**	317 ± 9.32	0.57 ± 0.02	2.27 ± 0.06
**sFlt-1**	322 ± 8.07	0.56 ± 0.01	2.31 ± 0.07
**sFlt-1+PIBF**	303 ± 6	0.54 ± 0.02	2.19 ± 0.06

## 4. Discussion

Because current treatments for PE are not continuously effective to improve maternal and fetal outcomes, there is an emphasis on drug discovery for the treatment of PE. The current therapeutics used for the development of severe preterm PE during pregnancy, prior to 34 weeks gestation, include intravenously-infused magnesium sulfate to slow disease progression and prevent maternal seizure, potent glucocorticoids to enhance fetal lung maturation, and anti-hypertensives to prevent stroke. Notably, we have utilized various anti-inflammatory agents, such as the T cell suppressor abatecept (Orencia) [34], tumor necrosis factor-α inhibitor etanercept [35], B lymphocyte-depleting agent rituximab [36], and IL-17RC [37] to decrease hypertension and circulating factors in RUPP rat model of PE that are also elevated during preeclampsia. Furthermore, all of these anti-inflammatory agents were able to effectively decrease blood pressure and inflammation in our RUPP model of PE, but have not been considered obstetrically safe for use in PE women. Considering our previously published data that progesterone is lower in PE women at out hospital compared to those with uneventful pregnancies, we believe that the utilization of progesterone in preeclamptic patients, or those who have developed clinical manifestations of preterm PE, is an area that requires additional focus [29]. We have shown that progesterone in the form of 17-OHPC administered after placental ischemia improved ET-1, uterine artery resistance, intrauterine growth restriction (IUGR), and blood pressure in rat models of PE [38,39], thereby further establishing proof of concept supporting that progesterone could be a beneficial treatment for PE. However, the mechanisms of action whereby progesterone supplementation could be beneficial for PE women was lacking. Ours and other labs have shown that PIBF, a product of progesterone signaling, lowers inflammation and vasoactive factors such as sFlt-1 and, thus, hypertension in the RUPP rat model of PE.

sFlt-1 is notoriously associated with PE, reduced renal function, and IUGR during pregnancy. We and others have shown that sFlt-1 induced hypertension is associated with reactive oxygen species (ROS) in pregnant rodents. Similar to sFlt-1, oxidative stress has an important role in the pathophysiology of PE. Therefore, in this study we tested the effect of sFlt-1 to cause mitochondrial function and the role of PIBF to normalize it and hypertension in a rat model of sFlt-1 induced hypertension during pregnancy. We demonstrated that PIBF lowers blood pressure in response to sFlt-1 and normalized mitochondrial function in the placenta of sFlt-1 induced hypertensive rats.

Mitochondrial oxidative stress has become an area of emphasis for many labs investigating the pathology of PE. We have shown a reduction of vascular endothelial mt respiration and increased mtROS to be caused by various inflammatory mediators, such as TNF-α, agonistic autoantibodies to the angiotensin II type I receptor, and CD4+T cells in pregnant rats. However, we do not know the effect of a broad anti-inflammatory agent on mt function and hypertension rat models of PE. Deleterious effects of free radicals include oxidative damage of biomolecules, initiation of lipid peroxidation, and cellular dysfunction, and are suggested to initiate maternal leukocyte activation and endothelial dysfunction in PE [40]. Typically, hypertension results from increases in the production of ROS in the vascular wall, thereby causing increases in oxidative stress and mediation of vascular diseases [41]. A major source of ROS production occurs in mitochondria and is produced by the complexes of the electron transport chain. Additionally, ROS is able to interact and inactivate nitric oxide to cause the endothelial dysfunction that occurs during PE, and, therefore, identifying agents that can protect mitochondria is necessary to prevent cellular damage. In this study, we demonstrate that PIBF released after progesterone signaling through its receptor can protect placental mitochondrial function and thereby improve overall organ function, thus resulting in lower blood pressure in the presence of sFlt-1.

McCarthy et al. showed that vascular mtROS and decreased respiration in HUVECs is caused by the release of soluble factors released in the circulation of PE women [42]. Sanchez-Aranguren et al. demonstrated an alteration in the mitochondrial bioenergetics that were induced from increases in sFlt-1 levels in HUVECS treated with plasma from PE women compared to normal pregnant women. [43]. Zhai et al showed that the proliferation of HUVECs was damaged and apoptosis was up-regulated when stimulated by sFlt-1, which further demonstrates that sFlt-1 contributes to endothelial dysfunction [44].

Endothelial dysfunction is a customary phenotype of PE characterized by vasoconstriction, reduced blood flow to organs, and the release of placental factors in response to placental ischemia [1,45,46,47]. Studies have previously indicated the importance of the maternal vascular endothelium in regulation vascular inflammation, and oxidative stress [48]. We have demonstrated that progesterone attenuated ET-1 secretion in HUVECS exposed to RUPP rat sera [29]. In this current study, we exposed HUVECs to sera from sFlt-1 induced hypertensive rats before and after supplementation with PIBF, and found a significant decrease in endothelial cell mtROS compared to those treated with NP+sFlt-1 serum. Therefore, these results coupled with our previous results indicate that progesterone could provide protection from soluble factors released in response to placental ischemia [29].

Studies have shown that placental overexpression of sFlt-1 is induced by hypoxia, suggesting that placental ischemia would promote sflt-1 expression and result in an imbalance between pro- and antiangiogenic factors in preeclampsia [49]. Furthermore, increased levels of sFlt-1 correlate with decreased NO formation in women with preeclampsia, which may additively work together to induce endothelial dysfunction. This data further implicates sFlt-1 as having detrimental effects in PE women and playing a key role in metabolic modulation and reprogramming in placenta and endothelium during pregnancy [43]. Importantly, we demonstrate PIBF to play a protective role against not only hypertension for placental and endothelial function during pregnancy.

Collectively, these findings further demonstrate that sFlt-1 causes hypertension and increases in mitochondrial dysfunction, but that these changes can be mitigated with progesterone. Therefore, the data suggests progesterone supplementation as a strategy to assist in the management of PE by correcting imbalances of hormones during pregnancy.

## 5. Conclusions

In conclusion, these results ultimately indicate that normal mitochondrial activity is important in normal pregnancies. Our data demonstrates that mt dysfunction could be normalized with certain therapeutic treatments, such as progesterone or progesterone products during pregnancy. Although the use of pharmacological inhibitors utilizing inflammatory cytokines or lymphocyte therapies are not well understood, our results reveal that administration of PIBF reduces hypertension, oxidative stress, and endothelial dysfunction during pregnancy and thereby indicates that there are exciting possibilities for obstetric use of progesterone in the future for preeclamptic pregnancies. Although our data demonstrates that PIBF can improve hypertension and mt function during pregnancy, the exact mechanism by which this occurs needs to be further examined.

## Data Availability

All data relevant to the study are included in the article.
